# The Relationship between Event-Based Prospective Memory and Ongoing Task Performance in Chimpanzees (*Pan troglodytes*)

**DOI:** 10.1371/journal.pone.0112015

**Published:** 2014-11-05

**Authors:** Theodore A. Evans, Bonnie Perdue, Michael J. Beran

**Affiliations:** 1 Language Research Center, Georgia State University, Atlanta, GA, United States of America; 2 Department of Psychology, Agnes Scott College, Decatur, GA, United States of America; University College London, United Kingdom

## Abstract

Prospective memory is remembering to do something at a future time. A growing body of research supports that prospective memory may exist in nonhuman animals, but the methods used to test nonhuman prospective memory differ from those used with humans. The current work tests prospective memory in chimpanzees using a method that closely approximates a typical human paradigm. In these experiments, the prospective memory cue was embedded within an ongoing task. Tokens representing food items could be used in one of two ways: in a matching task with pictures of items (the ongoing task) or to request a food item hidden in a different location at the beginning of the trial. Chimpanzees had to disengage from the ongoing task in order to use the appropriate token to obtain a higher preference food item. In Experiment 1, chimpanzees effectively matched tokens to pictures, when appropriate, and disengaged from the ongoing task when the token matched the hidden item. In Experiment 2, performance did not differ when the target item was either hidden or visible. This suggested no effect of cognitive load on either the prospective memory task or the ongoing task, but performance was near ceiling, which may have contributed to this outcome. In Experiment 3, we created a more challenging version of the task. More errors on the matching task occurred before the prospective memory had been carried out, and this difference seemed to be limited to the hidden condition. This finding parallels results from human studies and suggests that working memory load and prospective memory may have a similar relationship in nonhuman primates.

## Introduction

Prospective memory (PM) is the formation, storage, retrieval, and implementation of an intended future action – or, more succinctly, it is remembering to do something later. It is evident in many aspects of our lives, ranging from the mundane to the important, in which we must remember to do something at a later time. Every time one remembers to attach a file to an email before sending it or to take medication before going to sleep, some form of PM is at work. PM may be even more evident when it fails us, for example, when a man forgets that his wife asked him to pick up something from the market on the way home or when one fails to mail a bill payment before the due date. This psychological phenomenon has become well studied in the past three decades using both highly controlled laboratory tests and more naturalistic “real-world” scenarios [1], [2], [3]; for a review, see [4].

A common laboratory PM test begins with an experimenter instructing the participant to remember to perform a specific act upon the appearance of a particular word or word category on a computer screen, e.g. [5], [6]. For example, when the participant sees that word, he or she is supposed to remember to press a special key on the keyboard. Then, following a delay interval, the participant begins working on an unrelated task. The interval between the intention formation and onset of the task varies across studies from immediate presentation of the unrelated task, e.g. [7], to hours later, e.g. [8].

The unrelated task might be a lexical decision task in which the participant must quickly decide whether each string of letters presented on a computer screen is a real word or a non-word, e.g. [9]. Critically, when the participant sees the target word appear in the lexical decision task, he or she needs to remember to press the special key rather than sort it into one of the two categories. This type of task and other similar methods are used to examine a variety of questions about PM related to the underlying processes, influential variables, and development of this psychological phenomenon, e.g. [10], [11], [12]. For example, researchers study the degree to which PM retrieval cues are detected spontaneously or as a result of monitoring one’s environment, and this is done by analyzing differences in participants’ ongoing task performance as a function of whether or not they are asked to carry out a delayed intention, e.g. [12], [13], [14], [15], [16].

The laboratory PM test described above is commonly called an *event-based* task, and this is because the appropriate moment to perform the delayed intention is signaled by a particular event type (i.e., the appearance of the target word or word category). This is distinguished from the other major type of PM task, the *time-based* task, in which the opportunity to make the delayed response is defined by a certain clock time or a particular duration of elapsed time (e.g., remembering to press the F8 key ten minutes into an ongoing task) [17], [18]. Our research makes use of event-based prospective memory cues.

There has been a longstanding interest in the nature of past-oriented and future oriented memory in nonhumans animals, e.g. [19], and researchers have designed versions of these tasks suitable for testing nonhuman animals (hereafter animals) to explore whether PM is a uniquely human phenomenon. For instance, Wilson and Crystal [20] developed a rat (*Rattus norvegicus*) model of time-based PM by first teaching individual rats to perform a bisection task in which they indicated whether an experienced temporal duration was “long” or “short.” Rats were then taught that, after 90 minutes of performing the bisection task, they would have 30 minutes of access to food pellets, which could be obtained by poking their noses into a food trough. Across dozens of sessions conducted like this, the researchers found that, as time drew closer to the post-test meal, rats performed poorer on the bisection task and made more nose poke responses to the food trough. Wilson and Crystal [20] suggested that these rats formed a time-based PM to nose-poke in the trough and that shifting attentional resources toward executing this PM resulted in poorer performance in the ongoing bisection task. Wilson, Pizzo, and Crystal [21] performed an extension of this study in which the divide between the bisection task and the post-test meal occurred after a variable duration and was signaled by an auditory cue (i.e., an event-based task). The researchers found a similar decline in bisection task performance following the cue, again suggesting that the rats were anticipating the meal.

Nonhuman primates (hereafter primates) also have performed laboratory PM tests. Evans and Beran [22] presented capuchin monkeys (*Cebus apella*) and rhesus monkeys (*Macaca mulatta*) with an event-based PM task that was embedded within an ongoing two-choice discrimination task. In the ongoing task, monkeys used a joystick to repeatedly select the S+ (rewarded stimulus) from a pair of digital stimuli in order to earn individual food pellets. Occasionally, between these discrimination trials, monkeys saw a flashing visual stimulus that indicated that a “jackpot” of pellets was available at the end of the trial block (but not at the present time). The PM task was to remember, when appropriate, to touch a special stimulus at the end of the trial block rather than initiating the next block of discrimination trials. Monkeys learned to make the PM response when the visual cue occurred even with multiple discrimination trials still left to perform prior to the PM opportunity, and monkeys even initiated the PM response (by starting to move the cursor across the screen) before the special stimulus was visible on the response screen, suggesting that they were anticipating its appearance.

Research in this area also has been conducted with our closest living relative, the chimpanzee (*Pan troglodytes*). Beran, Perdue, Bramlett, Menzel, and Evans [23] tested a language-trained chimpanzee named Panzee in a PM task in which she had to remember to request a previously hidden food item when she encountered a lexigram token that represented that item. These lexigram tokens were visual symbols that each represented a specific food type. At the beginning of a session, Panzee chose from two food options the one she wanted to receive more immediately. An experimenter scattered this chosen option in an adjacent outdoor yard amongst an array of face-down lexigram tokens. Another experimenter sealed the non-chosen option in an opaque container that remained near the indoor test enclosure. Panzee could then enter the outdoor yard and forage for her chosen food option and (if she chose) view the lexigrams by turning over the tokens. Panzee typically ate all of her scattered items first. She then turned over tokens until she found the lexigram that matched the previously hidden item, and then returned to the indoor enclosure to exchange the token for the hidden item.

Perdue, Beran, Williamson, Gonsiorowski, and Evans [24] extended this research with Panzee (and three other chimpanzees) in which they replaced the ongoing foraging task with a more effortful quantity discrimination task that would more likely prevent continuous rehearsal of the PM target. Now, the chimpanzees performed a quantity judgment task in which they had to track the numbers of grapes an experimenter dropped into two different opaque containers, one of which they could have at the end of the trial. The PM test was whether the chimpanzees would remember, after completing several minutes of quantity judgment trials, to request the previously hidden food item (in this case, through a combination of attention-getting vocalizations and gestures). All four chimpanzees often remembered to do so and directed the experimenter to the location of the previously hidden item.

The above studies have demonstrated at least a rudimentary form of prospective memory in animals. As in many human laboratory PM tests, these experiments required animals to anticipate or perhaps even plan a future act, retain that plan or intention while engaged in other ongoing activity for some period of time, retrieve the PM at some point during (or following) the ongoing task, and finally execute the behavior at an appropriate time. However, human PM tasks are typically characterized by additional parameters that help set them apart from related, but different, psychological phenomena (e.g., retrospective memory, planning, working memory) [4]. One particular parameter that each of the above animal tests is lacking is complete integration of the PM and ongoing tasks. In a common human event-based prospective memory experiment (as described above), the cue to retrieve the PM intention appears as a regular part of the ongoing task [25]. This allows the cue to serve as a viable stimulus in both the ongoing task and the PM task. For example, a participant viewing a string of letters presented on a computer screen could either sort that stimulus as a word/non-word (as in a lexical decision task) or process that string as the cue to execute the delayed behavior (e.g., press a special key). In Wilson et al.’s [21] rat event-based PM study, the PM cue was an auditory stimulus that was irrelevant to the ongoing temporal bisection task. Similarly, in Evans and Beran’s [22] monkey PM study, the cue was a flashing visual stimulus that was irrelevant to the ongoing two-choice discrimination task.

The two previously conducted chimpanzee PM studies [23], [24] also did not fully integrate the PM and ongoing tasks, as the opportunity to execute the delayed behavior actually occurred just after the ongoing task in each study. In the human literature, these types of tasks are sometimes called “activity-based” tasks, e.g. [26], [27]. Even though these types of experiments have their real-world counterparts (e.g., remembering to call back a colleague after interrupting the call to attend a meeting), they are considered less sophisticated than common event-based assessments in which the two competing tasks are integrated as described above [28].

To assess whether our closest living relatives are capable of event-based prospective memory, as it is typically defined in the human literature, we designed a chimpanzee PM test that involved a PM cue that could be completely embedded within the ongoing task. As in Beran et al. [23] and Perdue et al. [24], the present test began with a chimpanzee watching an experimenter conceal a preferred food item in an opaque container. Also, as in Beran et al. [23], the PM task was to remember to request that hidden item when the chimpanzee encountered a lexigram token that represented that item (by exchanging the token with an experimenter near the concealed item). However, unlike in those previous tests, the ongoing task in the present study involved making conceptual judgments with regard to available lexigram tokens. In this ongoing task, an experimenter presented the chimpanzee one lexigram token at a time, and the chimpanzee was rewarded with a small treat for matching the token to a photograph depicting the item represented by the lexigram. However, once the chimpanzee received the token that represented the food item that was concealed at the beginning of the session, it should refrain from matching the token to one of the two patches of photographs and, instead, transport and exchange the token for the concealed item. Functionally, this is the same response required of human participants when they refrain from classifying the target stimulus as a word or non-word in the lexical decision task and, instead, perform the remembered response (e.g., pressing the spacebar when they see that particular word). Some chimpanzees succeeded in this version of a PM test, and therefore provided evidence of event-based PM in our closest living relative in an analogue of a sophisticated human PM test. These findings also highlight the chimpanzee as a useful model for testing other important questions such as the underlying processes or environmental contexts that support PM.

## Experiment 1

### Participants

We tested three language-trained chimpanzees including one male (Sherman, age 40) and two females (Lana, age 43; Panzee, age 28). All three chimpanzees were born in captivity at the Yerkes Regional Primate Research Center and had lived together at the Language Research Center for the last 23 years. All chimpanzees were housed together in the same building and spent time together in social groups daily, but they were tested separately. Chimpanzees were rewarded with preferred food treats for participating in the experiment. Chimpanzees also received a full diet of fruit, vegetables, and primate chow at multiple times each day and had ad libitum access to water (i.e., they were not food or water deprived for the purposes of testing). The chimpanzees were also provided various sources of enrichment when they were not testing, including (but not limited to) access to television, nesting materials, craft materials, toys, and outdoor climbing towers. Chimpanzees were never forced to participate, and they could choose when they wanted to work and when they wanted to rest. This study was carried out in strict accordance with the recommendations in the Guide for the Care and Use of Laboratory Animals of the National Institutes of Health. The protocol was approved by the Georgia State University Institutional Animal Care and Use Committee (Protocol Number: A13015).

All three individuals had been involved in language acquisition research from an early age in which they learned to associate geometric forms called lexigrams with different foods, locations, objects, and people [29], [30], [31], [32], [33]. These chimpanzees also participated in three prior studies involving lexigram tokens similar to the ones used here. In one study, these chimpanzees were tested for their ability to trade tokens with conspecifics for mutual gain [34]. In another study, they were assessed for self-control through their capacity to choose lexigram tokens representing highly preferred foods over immediately available, moderately preferred foods when the tokens could only be exchanged later for the foods they represented [35]. Most recently, all three chimpanzees were trained to use the lexigram tokens to request previously hidden food items, although only Panzee participated in the test phase of the experiment [23]. Also, as mentioned above, these chimpanzees participated in a prospective memory experiment in which they needed to remember to request a previously hidden food item, although in that case by using a combination of attention-getting vocalizations and gestures [24].

### Materials

**Figure 1:**
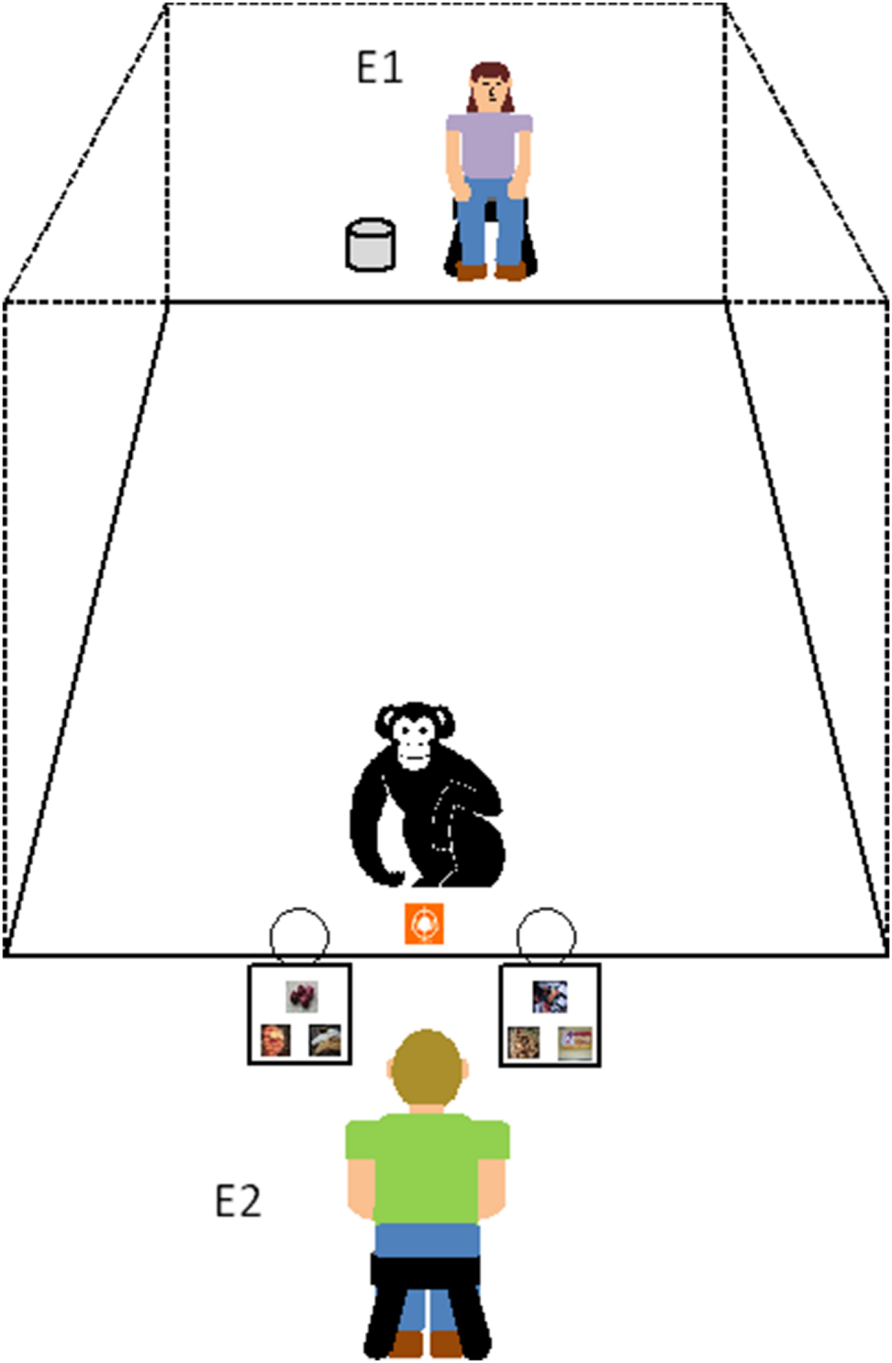
The experimental set-up and procedure for Experiment 1. At the beginning of each session, a chimpanzee watched as Experimenter 1 (E1) entered the test area and baited a metal bucket with a large preferred food item at the back of the test area. Experiment 2 (E2) then entered and sat in a chair between the two matching trays at the front of the test area and began the ongoing matching task by placing 3 unique photographs on each tray. E2 then passed the chimpanzee one lexigram token at a time, and the chimpanzee placed the token in the appropriate bowl (the one attached to the matching photograph) to earn small food rewards. But, when the chimpanzee received the token matching the previously hidden food item, it should instead carry that token to E1 to obtain the target food item.

We used lexigram tokens similar to those used in the prior studies mentioned above. They were 7.5 cm×7.5 cm×.5 cm white plastic squares with a laminated lexigram symbol affixed to one side. The lexigrams presented as tokens in this study represented the following food items: banana, bread, carrot, chow, coffee, *Coke*, juice, *M&M’s*, and orange. These were all lexigrams that each chimpanzee could accurately match to photographs of appropriate items, as assessed over years of vocabulary testing [36]. We also used a metal bucket (approximately 4 liters in volume) with a lid to conceal target food items. The target items were six preferred foods from the list above (banana, coffee, *Coke*, juice, *M&M’s*, and orange). Additionally, we used a matching apparatus that consisted of two separate plastic trays (see Figure 1). Attached to one end of each tray was a shallow 15-cm bowl, which could be slid into the chimpanzee’s test enclosure. Attached to the other end of each tray was a 30-cm plastic board to which laminated photographs could be adhered with *Velcro* (hook and loop) fasteners. We recorded all test sessions on a Sony Handycam digital video recorder.

### Procedures

#### General Procedure

The test always involved two experimenters. Experimenter 1 (E1) entered the test area with the metal bucket containing a preferred food item and carried the bucket to the back of the test enclosure (see Figure 1). E1 asked the chimpanzee to come to the back of the enclosure and then showed the chimpanzee the contents of the bucket by removing the lid, tilting the bucket towards the chimpanzee and lifting the item out with the other hand. E1 remained seated at the back of the enclosure with the covered bucket for the remainder of the test session. During the baiting process, Experimenter 2 (E2) remained outside of the test area so that he was naïve to the contents of the bucket. After the baiting process, E1 said “okay” to indicate that E2 could enter the test area. Then, E2 entered and sat in a chair between the two matching trays (see Figure 1) and prepared the matching task by placing 3 unique photographs on the experimenter’s side of each tray. E2 held each photograph close to the mesh for the chimpanzee to see before attaching it to the matching tray, and this process created a delay of approximately 1–2 minutes between the presentation of the food item by E1 and the beginning the ongoing task. These two sets of 3 photographs remained attached to each tray for the entire test session. E2 proceeded with each trial of the matching task by sliding the bowls (attached to the trays) into the chimpanzee’s enclosure and then passing one of the six lexigram tokens (that matched the photographs) to the chimpanzee. In each trial, the chimpanzee could place the token in either of the two bowls or could carry it to the back of the enclosure and slide it to E1. Placing the token in the appropriate bowl at the front of the test area earned the chimpanzee a small preferred treat (e.g., dried fruit, nut, small cookie or cracker). Placing the token in the incorrect bowl resulted in a 10-s timeout before the next trial. Transporting and passing an appropriate token to E1 earned the chimpanzee the hidden food item. If the chimpanzee passed an inappropriate token to E1, then E1 would keep the token and tell the chimpanzee that there was no (*item x*) in the bucket (and the chimpanzee either returned to the front of the enclosure on its own or was asked to do so by E2). In this case, the item was not revealed to the chimpanzee.

#### Training

Because chimpanzees had experience using lexigram tokens to request concealed food items, training primarily involved familiarizing the chimpanzees with the lexigram-to-photograph matching task that was the ongoing task in this experiment. We did this training in isolation of the prospective memory task so as to avoid actually training the chimpanzees to integrate the two tasks. Thus, during these training trials, E1 did not bring a bucket or target food item into the test area, and she did not sit at the back of the test enclosure. The first session of training involved just one photograph attached to each tray for each trial, and we presented four 4-trial blocks with the same pair of photographs (and corresponding lexigrams) within each block to ensure that the chimpanzees understood the matching procedure. Next, we presented the chimpanzees with 15-trial sessions of mixed pairs of photographs/lexigrams until each individual reached a criterion of 12/15 correct for two consecutive sessions. We then increased the number of photographs displayed on each tray to two and continued this training until a chimpanzee reached the criterion of 12/15 correct in one session. Finally, we increased the number of photographs on each tray to three and trained the chimpanzee until it again met that criterion. Thus, at the end of training, the chimpanzees could take a token that was passed to them and place it into one of two bowls, each of which had three photographs attached to it. Success in training meant proficient sorting of lexigrams tokens into these bowls dependent on the photos affixed to them.

Because the chimpanzees had not used these lexigram tokens to request actual food items for over a year (since the end of [23]), and because they had just been encouraged to use them in an entirely new context (the matching task), we next presented each chimpanzee with a short series of sessions to reacquaint them with the token request procedure within this new context. These sessions consisted of three consecutive 6-trial blocks in which the same target food item was available until the chimpanzee disengaged from the matching task and used an appropriate token to request the target food item from E1 at the back of the enclosure. Following each block that a chimpanzee failed to obtain the target item, E1 reminded the chimpanzee of the bucket’s contents by removing the lid and showing the item inside. Once the chimpanzee requested the target item with the appropriate token, E1 delivered the item in entirety and the bucket remained empty for the rest of the session. We required each chimpanzee to do this successfully once within the first trial-block while maintaining above-criterion matching performance before moving on to the test phase. Thus, by the end of training, the chimpanzees had learned both components of the task and began to successfully integrate them.

#### Testing

Each chimpanzee participated in 12 test sessions involving the General Procedure described above. We conducted each session on a separate day and we typically tested chimpanzees two days per week. Unlike in the training phase, each test session involved one matching trial per photograph (and corresponding lexigram) for a total of 6 matching trials. Additionally, if the chimpanzee successfully disengaged from the matching task and used the appropriate lexigram token to request the hidden food item, we repeated the matching trial involving that token at the end of the session. These instances served as control trials in which no target food item was available in the bucket, because the chimpanzee already exhausted it (see also [37] regarding use of this trial type with human participants in in the context of *suspended intentions*). If, however, the chimpanzee did not remember to request the target item at the appropriate time, we queried the chimpanzee about the contents of the bucket at the end of the session. E1 drew the chimpanzee’s attention, held up the bucket, and asked “what’s in here?” while pointing to the bucket (and if necessary, gestured to a wall-mounted lexigram keyboard to encourage a response). Thus, any disengagement from the sorting task during a session was spontaneously generated by the chimpanzee without any explicit cue to do so other than seeing the token with the lexigram representing the item in the bucket behind them.

The target item and accompanying set of photographs/lexigrams was determined randomly by E1 prior to each test session. The presentation order of lexigram tokens was determined pseudo-randomly to ensure that an equal number of sessions involved target tokens that occurred in the first and second half of sessions. This also prevented chimpanzees from using the elapsed interval since baiting as a cue to avoid making erroneous token exchanges during the control trials that concluded test sessions. Also, to reiterate, the experimenter who was working with the chimpanzee during the sorting task did not know what item was in that bucket, and therefore could not provide any inadvertent cues as to when the chimpanzee should disengage sorting and instead take the token and walk to the back of the testing area to exchange it with the other experimenter.

## Results

### 

#### Training

All three chimpanzees required 2 to 4 sessions to meet or exceed the training criterion (12/15 correct for two consecutive sessions) when matching a lexigram token to a photograph when each tray displayed one photograph. Each chimpanzee also required one additional session to meet the training criterion (12/15 correct in one session) involving two or three photographs (2 total sessions each). Each chimpanzee also required 4 to 6 additional sessions to exchange a lexigram token for a target food item within the first block of an integrated matching/memory training session.

#### Testing

Table 1 summarizes each chimpanzee’s ongoing matching task performance and PM task performance, as coded by E2 in real-time during test sessions. We confirmed the reliability of these data by having an independent observer, who was not part of this study and was unaware of the hypotheses or goals of the study, code 50% of test sessions from video (Kappa = 0.975, *p*<0.001). Each chimpanzee continued to match lexigram tokens to photograph sets at high accuracy in the test phase (Lana: 100% correct; Panzee: 97.2% correct; Sherman: 97.44% correct). Each chimpanzee also disengaged from the matching task and exchanged an appropriate token for the target food item in most sessions (Lana: 75% of sessions; Panzee: 75% of sessions; Sherman: 83.33% sessions). Additionally, chimpanzees never attempted to obtain the target food item using the same token in control trials at the end of the session, once the food item had been already exhausted. Instead, on the second presentation of that token they sorted it into the appropriate bowl. This pattern of responding, with regard to what tokens were taken to the bucket and what tokens were sorted, differed from a chance distribution, according to individual 2×2 Fisher Exact Probability tests (all *p*<.001).

**Table 1:** Chimpanzees’ ongoing task and memory task performance in Experiment 1.

	Ongoing matching task			
	Correct	Incorrect	Total	% Correct
Lana	50	0	50	100
Panzee	64	2	66	96.97
Sherman	76	2	78	97.44
	**Prospective memory exchange task**			
	***(Sessions involving successful token exchange for a target item)***			
	**Target Present**	**Target Exhausted**	**Total**	**% Correct**
Lana	9	0	12	75
Panzee	9	0	12	75
Sherman	10	0	12	83.33
	***(Tokens passed to an experimenter to obtain a target item)***			
	**Appropriate**	**Inappropriate**	**Total**	**% Correct**
Lana	9	23	32	28.13
Panzee	9	7	16	56.25
Sherman	10	0	10	100

Of the sessions in which chimpanzees passed a lexigram token to E1 at the back of the enclosure, the appropriate token was sometimes, but not always, their first and only attempt (see also Table 1). Lana passed an appropriate token to E1 on her first exchange attempt more often than expected by chance levels (4 of 9 sessions; Binomial test, one-tailed exact *p* = 0.048; chance probability = 1 in 6 possible tokens or 0.167). However, Lana passed other inappropriate tokens to E1 beyond her first exchange attempts in 6 sessions, 5 of which involved her passing every token to E1 until she obtained the target item. Panzee was somewhat more selective. She passed E1 an appropriate token on her first exchange attempt in 5 of 9 sessions (Binomial *p* = 0.009) and passed one or two inappropriate tokens total to E1 in each of 4 sessions. Sherman was most accurate and never attempted to exchange an inappropriate token for the target item (10 of 10 sessions; Binomial *p*<.001). Thus, there were individual differences in how the chimpanzees performed with regard to token exchange at the location of the bucket.

## Discussion

From this experiment, we learned that at least some chimpanzees can exhibit prospective memory in a task analogous to those used with adult humans in the laboratory. The critical difference between this task and the previous tasks we have presented to the chimpanzees is that the prospective memory cue was embedded within a concurrent task that involved using the token symbols in a way that conflicted with the prospective memory target behavior.

This symbolic task was rewarded for good performance, thereby ensuring that the chimpanzees were somewhat motivated to perform that task as well as (if possible) retrieve the hidden food item in the bucket. Yet all three chimpanzees disengaged from the ongoing task at the appropriate moment in most sessions of this experiment. However, only Sherman limited his choices to execute the delayed behavior to appropriate opportunities to do so. Lana, and to a lesser degree Panzee, seemed to focus her efforts on obtaining the hidden food item because she forwent many smaller (but still preferred) treats while attempting to exchange inappropriate tokens for the larger hidden item. This behavior may have been inadvertently encouraged by our experimental design in which all sessions involved a large, preferred target food item. Because the delayed behavior was always required, it also may have become the primary activity for the chimpanzees more so than the sorting task (at least for Lana), and may even have come to be a more rote response in terms of remembering to exchange the token.

In Experiment 2, we conducted a similar test, but now there were sessions in which chimpanzees did not have to remember to ask for a hidden target food item in addition to trials like those in Experiment 1. In this new trial type, we placed an entirely visible target item in front of the chimpanzee and therefore removed the need for the chimpanzees to remember to take the token to another area when it was presented. This allowed us to assess whether the chimpanzees would perform any differently when they sometimes had to remember to exchange the token but other times did not. We expected chimpanzees to continue to remember to exchange the token for the hidden target item, although possibly at a lower rate, even when prospective memory was not required in every daily session.

This new condition also allowed us to look at whether performance on the matching task differed as a function of the PM memory load. Researchers sometimes perform this type of analysis in human prospective memory studies to examine the degree to which PM retrieval cues are detected spontaneously or as a result of monitoring one’s environment, e.g. [12], [13], [14], [15], [16]. A difference in ongoing task performance as a function of whether or not the participant is required to carry out a delayed intention is sometimes taken to mean that the participant is monitoring for PM retrieval cues. Because prospective memory is a sophisticated behavioral/cognitive phenomenon, especially for a chimpanzee, we predicted that chimpanzees would need to monitor their environment for the appearance of an appropriate token in sessions requiring PM and this would result in slightly lower token matching performance in such sessions compared to sessions with a visible target item.

## Experiment 2

### Participants

In this experiment, we again tested Lana, Panzee and Sherman.

### Materials

We used the same materials as in Experiment 1, but with one exception. We introduced a second metal bucket, identical to the one used in Experiment 1, for the purposes of this experiment. During some sessions, this bucket was positioned on its side, without a lid, underneath E2’s seat so that the chimpanzee could clearly see its contents throughout the trial.

### Procedures

#### General Procedure

This experiment included two session types, *Hidden* and *Visible*, which differed with regard to whether the target food item was concealed or not during the test session. Hidden sessions were very similar to the test sessions of Experiment 1, with the exception that, at the beginning of each session, E1 showed the chimpanzee an empty, lidless bucket and placed that bucket on its side under E2’s seat, with the open top of the bucket facing the chimpanzee. E1 then proceeded with baiting the bucket at the back of the test enclosure as in Experiment 1. Visible sessions differed from Hidden sessions only in that the lidless bucket placed under E2’s seat was baited with a large target food item, whereas the bucket placed at the back of the enclosure was left empty. In both visible and hidden conditions, subjects still had to pass the appropriate token to the experimenter when it became available in the matching task in order to obtain the item (see training section below for more detail).

Both session types also differed from Experiment 1 sessions in that, at the beginning of each ongoing task trial, E2 drew the chimpanzee’s attention to the bucket under his seat by pointing to it and saying “Don’t forget what’s down there.” Thus, in Visible sessions, there was still a large preferred food item to anticipate eating, but chimpanzees did not necessarily have to remember to ask for that item, since it was always visible and since the experimenter often reminded them of its presence.

#### Training

For the purposes of this experiment we needed to teach the chimpanzees a new response option so they could obtain the visible food item that was sometimes available at the front of the test enclosure (Visible trials). This response involved sliding the token toward E2 and the open bucket at the front of the enclosure rather than sorting that token. We began this training with a single session in which we baited the open bucket with a large food item and instructed the chimpanzees on how to obtain it. E2 passed an appropriate token to the chimpanzee and then pointed to the visible food and told the chimpanzee to “push out your token” (a statement often used to recruit a chimpanzee’s help in cleaning up after a research or husbandry event). Each time the chimpanzee passed the token to E2, E2 would give the chimpanzee a portion of the food item. This was repeated until the food was exhausted (5 or 6 trials per chimpanzee).

We next conducted a single session in which E1 baited the open bucket at the front of the test enclosure with a large food item and then E2 presented the chimpanzee with a block of matching trials in which the visible food item was represented by one of the lexigram tokens *but not* by one of the photographs on the matching trays. Thus, there was only one appropriate response for each token trial – either place it in one of the bowls with photographs or pass it forward to E2. In this session, E2 did not instruct the chimpanzee in any way, but instead allowed the chimpanzee to decide what to do with each token.

Finally, we conducted sessions in which the visible target food item was represented by one of the lexigram tokens *and* by one of the photographs on the matching trays and thus required the chimpanzees to actively disengage from the matching task and use the appropriate token to obtain the visible food item. These sessions consisted of two 6-trial blocks. To encourage the chimpanzees to pass the appropriate token to E2, once an error was made (a token was matched to photographs when it should have been passed forward to E2), the target item was taken away and replaced with an item of lower preference value. We conducted sessions in this way until each chimpanzee obtained the target item on the first opportunity to do so in two consecutive sessions, while maintaining ≥80% matching accuracy with non-target lexigrams.

#### Testing

Each chimpanzee participated in 10 Hidden sessions and 10 Visible sessions involving the general procedure described above. Unlike in Training, test sessions each consisted of a single block of six matching trials, and thus, chimpanzees had only one opportunity to obtain the target food item per session. As in Experiment 1, only one session was conducted on a given test day, and chimpanzees were tested two days per week. Session type, target item, and the accompanying photographs/tokens were all determined randomly prior to each session. The presentation order of lexigram tokens was again determined pseudo-randomly to ensure that an equal number of sessions involved target tokens that occurred in the first and second half of sessions.

## Results

### 

#### Training

All three chimpanzees learned to pass lexigram tokens to E2 in training session 1, in which there were no matching trays available, and in training session 2, in which matching trays were available but did not include a photograph of the visible target food item. In the following training sessions, in which matching trays were available and a photograph of the target item was present on one of the trays, Sherman and Panzee required 6 and 8 sessions, respectively, to meet the final training criterion (2 consecutive sessions in which they passed forward the appropriate token to E2 on the first opportunity, while maintaining ≥80% matching accuracy). Lana, however, never reached this criterion, as she persisted in passing most tokens to E2 when she should have been matching them to photographs. Therefore, Lana did not proceed to the testing phase.

#### Testing

Table 2 summarizes each chimpanzee’s ongoing matching task performance and PM task performance, as coded by E2 in real-time during test sessions. We confirmed the reliability of these data by having an independent observer code 50% of test sessions from video (Kappa = 0.956, *p*<0.001). As in Experiment 1, each chimpanzee disengaged from the matching task and exchanged an appropriate token for a *hidden* target food item in most sessions (Panzee: 70% of sessions; Sherman: 70% of sessions). However, chimpanzees never attempted to obtain the target food item in control trials at the end of each these sessions (those trials in which the token for the PM item was re-presented). This pattern of responding differed from a chance distribution, according to individual 2×2 Fisher Exact Probability tests (both *p* = .003). Additionally, neither chimpanzee attempted to exchange an inappropriate lexigram token for a hidden target item in these sessions.

**Table 2:** Chimpanzees’ ongoing task and memory task performance in Experiment 2.

	Ongoing matching task							
	Hidden sessions				Visible sessions			
	Correct	Incorrect	Total	% Correct	Correct	Incorrect	Total	% Correct
Panzee	64	4	68	94.12	67	0	67	100
Sherman	68	0	68	100	66	1	67	98.51
	**Prospective memory exchange task**							
	***(Sessions involving successful token exchange for a target item)***							
	**Hidden sessions**				**Visible sessions**			
	**Target Present**	**Target Exhausted**	**Total**	**% Correct**	**Target Present**	**Target Exhausted**	**Total**	**% Correct**
Panzee	7	0	10	70	8	0	10	80
Sherman	7	0	10	70	8	0	10	80
	***(Tokens passed to an experimenter to obtain a target item)***							
	**Hidden sessions**				**Visible sessions**			
	**Appropriate**	**Inappropriate**	**Total**	**% Correct**	**Appropriate**	**Inappropriate**	**Total**	**% Correct**
Panzee	7	0	7	100	8	0	8	100
Sherman	7	0	7	100	7	1	8	87.5

Chimpanzees performed similarly in sessions involving a *visible* target item at the front of the test enclosure. Both individuals disengaged from the matching task and exchanged the appropriate token for the visible target item in 80% of sessions, and chimpanzees rarely attempted to obtain the visible target item in control trials at the end of the session (Panzee: 0%; Sherman: 10%). Again, this pattern of responding differed from a chance distribution, according to individual 2×2 Fisher Exact Probability tests (Panzee: *p* = .001; Sherman: *p* = .005). Chimpanzees rarely attempted to exchange an inappropriate lexigram token for a previously visible target item in these sessions (Panzee: 0%; Sherman: 1%).

Chimpanzees’ token trading behavior in target trials did not differ between the two session types (both chimpanzees: 70% correct Hidden vs. 80% correct Visible; Fisher Exact test *p* = 1). As in Experiment 1, Panzee and Sherman matched lexigram tokens to photograph sets at high accuracy, regardless of session type, and a comparison of performance in those sessions types indicated no difference (Panzee: 94.12% Hidden vs. 100% Visible; Fisher Exact test, p = .12; Sherman: 100% Hidden vs. 98.51% Visible; Fisher Exact test, p = .5).

## Discussion

In this experiment, in which there was not always a prospective memory requirement, the chimpanzees continued to succeed overall in the task. The PM requirement of the Hidden condition did not significantly reduce chimpanzees’ ability to obtain the target food item in comparison to the Visible condition in which the visible target item served as a constant reminder to execute the delayed behavior. The PM requirement also did not hamper chimpanzees’ ability to accurately perform the ongoing task.

In the human PM literature, a decline in ongoing task performance is sometimes interpreted as a sign that cognitive resources are being shifted from performance of the ongoing task to monitoring for potential PM retrieval cues [12], [13], [14], [16]. Therefore, one might interpret the results of this experiment to mean that chimpanzees were not monitoring for PM cues (appropriate lexigram tokens), and instead were detecting them spontaneously. However, it is also possible that our ongoing matching task was not sensitive enough to reflect the existence of monitoring. Indeed, all chimpanzees were near ceiling level of performance on the matching task, and it is possible that it was easy enough for chimpanzees to perform this ongoing task and monitor for the PM cue without negatively influencing their performance of either task. This possibility provided the motivation for our third experiment, which involved a more challenging version of the ongoing task. We made the matching task more difficult by inserting a delay between presentation of the match photographs and the lexigram token, during which time all of these stimuli were masked. Therefore, the chimpanzee had to engage working memory during each trial of the ongoing task while maintaining the prospective memory.

## Experiment 3

### Participants

We began this experiment with the same participants as in Experiment 2 (Panzee and Sherman). However, during the course of the experiment, Panzee died from complications related to a chronic health condition (for which she was receiving regular veterinary care). Therefore, only Sherman completed the experiment and only his data are reported here.

### Materials

We used the same materials as in Experiment 2 except for one addition. Here we also used a set of two opaque canvas covers to mask the match photographs during the delay period of the ongoing task trials (see below for more details).

### Procedures

#### General Procedure

The task in this experiment began exactly as in Experiment 2 (with E1 baiting either the front or back bucket). E2 then began the first trial of the ongoing task, as usual, by displaying three photographs on each tray. Next, E2 held up a lexigram token in front of the chimpanzee for 2 to 3 seconds and then placed the token face-down on his lap. E2 then placed a canvas cover over each tray and looked at the floor for 10 seconds (to avoid cuing the chimpanzee). Finally, E2 pointed to the bucket under his seat while saying to the chimpanzee “Don’t forget what’s down there,” and then slid the token (face-down) to the chimpanzee. As in Experiment 2, the chimpanzee could place the token in either bowl attached to the matching trays, pass the token forward to E2 (to request a visible food item under E2’s chair), or carry the token to E1 (to request a hidden item from the bucket at the back of the enclosure). E2 then repeated the above steps for each remaining matching trial.

#### Training

Note that Sherman began training on a slightly different Experiment 3 method in which he did not see the lexigram token until after the delay period. Thus, the first time he saw the lexigram was when he flipped over the token after the delay period. Sherman did not seem to attend to the photograph arrays before they were covered in this version of the task, so we modified the method so that he would see briefly the lexigram token before the photographs were covered (as described in the General Procedure section).

The training phase of this experiment prepared chimpanzees for the delayed matching task that would replace the simultaneous matching task of the previous experiments. The first training sessions followed the general procedure for the ongoing task (described above) but involved only a minimal delay. As soon as E2 covered the photographs on the trays, he slid the face-down token to the chimpanzee. We conducted 6 trials in each of these sessions so that each photograph was the correct match stimulus in only one trial. We required chimpanzees to make at least 5 of 6 correct token-to-photograph matches in three consecutive sessions before increasing the delay during which the photographs were covered. In the following sessions, E2 waited 5 seconds between covering the photographs and passing the chimpanzee the lexigram token. We required chimpanzees to match correctly in at least 5 of 6 trials of 2 consecutive sessions involving a 5 second delay. Next, we increased the delay interval to 10 seconds and again required chimpanzees to match correctly in at least 5 of 6 trials of 2 consecutive sessions before moving on.

Subsequently, we slightly modified the procedure of the ongoing task to ensure that chimpanzees could not solve the task by positioning or orienting their bodies or gaze towards the correct tray during the full delay interval (rather than using memory, as we intended). In these sessions, E2 passed each token to the chimpanzee through one of the front corners of the enclosure so that the chimpanzee had to leave the area immediately in front of the matching trays in order to retrieve the token (approximately 1 to 2 meters from the starting position). We required each chimpanzee to match 5 of 6 trials accurately in 4 consecutive sessions to complete the delayed matching training phase.

Finally, before beginning the testing phase, we reintroduced target food items into sessions to be certain the chimpanzees had not forgotten this component of the task. We presented each chimpanzee with one session of each type (Hidden and Visible) using the general procedure described above.

#### Testing

Test sessions were similar to those of Experiment 2 except that the ongoing task consisted of the 10-second delayed matching task introduced during the training phase of this experiment. As in Experiment 2, we presented chimpanzees with an equal number of sessions in which E1 placed the target food item in the lidless bucket at the front of the enclosure or in the bucket with the lid at the back of the enclosure (10 sessions per condition). We also conducted a smaller number of *No-Target* sessions (6) in which there was no target food item in either location. We included these sessions to demonstrate that chimpanzees would not use lexigram tokens to ask for target food items in sessions in which those items did not exist.

As in the previous experiments, we conducted only one session per test day, and we tested chimpanzees two days per week. Prior to each session, we randomly determined the session type, target item, and accompanying photographs/tokens. We again presented the lexigram tokens in pseudo-random order to ensure that an equal number of sessions involved target tokens that occurred in the first and second half of sessions.

## Results

### 

#### Training

Each chimpanzee required the minimum number of sessions to reach the training criterion for the delayed matching task when the delay period was 0 s, 5 s, and 10 s (5 of 6 trials correct in 3, 2, and 2 consecutive sessions, respectively). Each chimpanzee also required the minimum number of sessions to reach the training criterion when they had to retrieve the token from the corner of the enclosure before placing it in a matching tray (5 of 6 trials correct in 4 consecutive sessions).

#### Testing

Table 3 summarizes Sherman’s ongoing matching task performance and PM task performance, as coded by E2 in real-time during test sessions. We confirmed the reliability of these data by having an independent observer code 50% of test sessions from video (Kappa = 0.898, *p*<0.001). As in Experiment 2, Sherman’s token PM performance did not differ between Hidden and Visible sessions in this experiment. In these sessions, Sherman disengaged from the matching task and exchanged an appropriate token for a target food item in most sessions (90% of sessions of each type). Also, he never attempted to obtain the target food item in control trials at the end of either of these session types (those trials in which the token for the PM item was re-presented). His pattern of responding in each session type (with regard to what tokens were passed toward the buckets and what tokens were sorted) differed from a chance distribution, according to separate 2×2 Fisher Exact Probability tests for each session type (both *p*<.001). Additionally, Sherman never attempted to exchange an inappropriate lexigram token for a hidden or visible target item in test sessions. Moreover, Sherman never attempted to exchange a token to either experimenter in No-Target sessions (in which there was never an available target item). Instead, in these sessions, he always placed tokens into the matching trays.

**Table 3:** Sherman’s ongoing task and prospective memory task performance in Experiment 3.

	Ongoing matching task													
	Hidden sessions					Visible sessions					No-Target sessions			
	Correct	Incorrect		Total	% Correct	Correct	Incorrect		Total	% Correct	Correct	Incorrect	Total	% Correct
Before	15	8		23	65.22	13	7		20	65	n/a	n/a	n/a	n/a
After	26	3		29	89.66	29	5		34	85.29	n/a	n/a	n/a	n/a
Total	41	11		52	78.85	42	12		54	77.78	30	6	36	83.33
	**Prospective memory exchange task**													
	***(Sessions involving successful token exchange of a target item)***													
	**Hidden sessions**					**Visible sessions**						**No-Target sessions**		
	**Target** **Present**		**Target** **Exhausted**	**Total**	**% Correct**	**Target** **Present**	**Target** **Exhausted**	**Total**		**% Correct**	**Target** **Present**	**Target** **Exhausted**	**Total**	**% Correct**
Total	9		0	10	90	9	0	10		90	0	0	0	0
	***(Tokens passed to an experimenter to obtain a target item)***													
	**Hidden sessions**					**Visible sessions**						**No-Target sessions**		
	**Appropriate**	**Inappropriate**		**Total**	**% Correct**	**Appropriate**	**Inappropriate**	**Total**		**% Correct**	**Appropriate**	**Inappropriate**	**Total**	**% Correct**
Total	9	0		9	100	9	0	9		100	n/a	n/a	n/a	n/a

Note: *Hidden* and *Visible* refer to session types in which the target food item was hidden in a closed bucket at the back of the test enclosure throughout the session or was entirely visible in an open bucket at the front of the test enclosure throughout the session. *Before* and *After* refer to trial positions that occurred before or after the target trial involving the lexigram token that matched the target food type.

As in Experiment 2, Sherman’s PM performance in target trials did not differ between the Hidden and Visible sessions (90% both session types; Fisher Exact test, *p* = 1). Also, Sherman’s overall token-to-photograph matching performance did not differ between Hidden, Visible, and No-Target sessions (78.85%, 74.07%, and 83.33% correct respectively; *χ* ^2^ = .437, *df* = 2, *p* = .804). However, Sherman did seem to commit a larger percentage of errors in matching trials conducted before the target trial (i.e., the trial involving a token that matched the identity of the large target food type) than after the target trial in both Hidden and Visible sessions (Hidden: 65.22% correct before vs. 89.66% correct after; Visible: 65% correct before vs. 85.29% correct after). To confirm this apparent effect for each session type, we conducted 2×2 Fisher Exact Probability tests, and we found that position (before/after) by outcome (correct/incorrect) significantly influenced the distribution of Sherman’s responses in the Hidden condition (*p* = .044), but not in the Visible condition (*p = *.10).

We also conducted two post-hoc analyses to rule out alternative explanations for the results of Experiment 3. First, we assessed whether the effect of trial position on matching accuracy in the hidden condition could be the result of a practice effect. We assessed this by calculating Sherman’s matching errors as a function of trial number in the No-Target sessions (in which there was no target item). Sherman’s errors did not appear to relate to how early in the sequence the matching trial occurred in these sessions (*r*(4) = 0.0, *p* = 1.0; Position 1: 2 errors, Position 2: 1 errors, Position 3: 0 errors, Position 4: 0 errors, Position 5: 1 error, Position 6: 2 errors). The same analysis demonstrated that Sherman’s errors did not decrease over trials as a result of a changing chance performance level that resulted from using the same six photographs for all six trials in each session, as described in the Experiment 1 methods section. Second, we examined whether chimpanzees’ lack of token exchanges in control trials at the end of Hidden sessions (i.e., the final trial of each session that represented the target lexigram token after the target food item had been exhausted) could be explained by within-session memory decay. We assessed this by calculating the number of instances in which chimpanzees failed to exchange the target token for the target food item as a function of trial position within Hidden sessions of all three experiments. Such instances were not systematically related to how late in the trial sequence the target token appeared (*r*(4) = 0.057, *p* = 0.914; Position 1: 1 error, Position 2: 2 errors, Position 3: 4 errors, Position 4: 2 errors, Position 5: 5 errors, Position 6: 0 errors).

## Discussion

With a presumably more challenging ongoing task that involved working memory resources, Sherman’s PM performance (i.e., his ability to remember to pass the appropriate token to E1 to obtain the hidden food item) did not suffer in this experiment. However, his performance on the delayed matching ongoing task was notably below ceiling level (unlike in the task used in Experiments 1 and 2). More specifically, he erred most on delayed matching trials that occurred before the target trial of Hidden sessions (i.e., before the trial involving the token that matched the hidden food item). This suggests that, during those early ongoing task trials, when he had not yet retrieved the target item, he was committing some degree of cognitive resources to remembering to make the PM response. Because this same effect was not significant for the Visible sessions, in which Sherman did not necessarily have to remember to make that response (since the experimenter regularly drew his attention to a visible target item), one cannot necessarily attribute the more frequent pre-target matching errors in the Hidden condition to anticipation of consumption of a large preferred food item. Also, because there was no effect of trial position on matching performance in No-Target sessions, Sherman’s performance in Hidden sessions could not be attributed to a within-session practice effect or attributed to a side-effect of a changing chance performance level. Rather, the effect seemed to be specific to having to remember to retrieve the target item when its token appeared.

## General Discussion

Chimpanzee prospective memory appears to be functionally similar to that of human prospective memory. In this series of experiments, chimpanzees recognized something they needed to remember. Specifically, they saw a food item they wanted, and they remembered what it was (and where it was, although we consider this to be a trivial aspect of the present experimental task compared to other past demonstrations of chimpanzee spatial memory; e.g., [38], [39]). The question of interest was whether they would remember to retrieve those food items at the appropriate time using a response mode (token exchange) that was also part of an ongoing task activity that engaged the chimpanzees. They did, providing evidence that they, like humans, could disengage from an ongoing task when a specifically highlighted stimulus in that task (the token for the hidden food) was presented. Critically, the chimpanzees showed that once they had retrieved that hidden item, their next exposure to that same token in the same test session instead led to it being used in the ongoing task. Because chimpanzees’ occasional failures to exchange target tokens for available target food items did not increase gradually across trials, this performance pattern could not be explained by within-session memory decay. Thus, the PM cue was selective in its production of the PM response, and the chimpanzees modified their behavior according to whether they still needed to recognize and use that cue to obtain the hidden food or not.

We had expected that Experiment 2 would produce a difference in performance depending on whether there was a PM “load” (the Hidden condition) or not (the Visible condition), but this did not occur. In human PM research, such manipulations often differentially affect PM performance, e.g. [12], [13], [14], [16]. However, we likely did not have an ongoing task that was sufficiently difficult. The ongoing matching task likely required little cognitive processing to perform, at least in terms of working memory resources. Experiment 3 appeared to remedy this issue (with Sherman), and in that case, he seemed to make more errors prior to implementing the prospective memory than after. This result appears similar to that reported for rats [40]. Critically, this was not due simply to a preferred food still being available for retrieval, as this effect was stronger when the target item was in the opaque container (which required that Sherman remember to retrieve it) than when it was visible and Sherman was reminded about it each trial (no PM needed). However, this is only a preliminary result, with one chimpanzee, but it suggests that chimpanzee prospective memory may sometimes require cognitive resources that make ongoing task performance more difficult as has been reported for humans, e.g. [41]. More research will be required to better understand this relationship, and to better assess the roles of spontaneous retrieval and monitoring in chimpanzee prospective memory.

Finally, it is important to note that, as in human prospective memory, e.g. [13], [16], there were individual differences in chimpanzee performance. In Experiment 1, Lana appeared to take a very different approach to the task than Sherman and Panzee. It appeared that she was much more concerned with getting the hidden item than in also performing the ongoing task, as she attempted to trade every token on some occasions. This is not likely due to her inability to remember what is in hidden containers, as she has been very proficient in other recent tasks of item memory for hidden objects, e.g. [42]. Rather, it appears that Lana struggled to accommodate the different response modalities that were available to her to perform both tasks (the ongoing matching task, and the PM task) at the same time. Panzee and Sherman were more proficient, perhaps as a result of their different rearing histories with lexigrams (see [32]), or perhaps as a result of some other aspect of their memory or cognitive control abilities. These individual differences often exist in studies with small numbers of animals in cognitive tests, and are important to remember when thinking about the broader generality of the results. Thus, we conclude that chimpanzees have the capacity for prospective memory in tasks mimicking those used with humans, but that they also show the variability seen in humans, and perhaps may not show true functional equivalence with human prospective memory. Certainly, there is at present no way to determine whether chimpanzee prospective memory has any of the conscious qualities that human PM has such as a sense of mental time travel, e.g. [43], or an anticipation of the future *as one will experience it oneself* (autonoesis; see [44]). It may not. But chimpanzee PM certainly meets many of the objective defining criteria [13], [16], [45], and therefore provides insights into the evolutionary foundations of this capacity for humans.
